# Network pharmacology and molecular docking to explore the active compounds and mechanisms of Jerusalem artichoke for treating diabetes

**DOI:** 10.1097/MD.0000000000041587

**Published:** 2025-10-17

**Authors:** Zhihua Guo, Xueting Zhu

**Affiliations:** a School of Biological and Food Engineering, Suzhou University, Suzhou, Anhui, China.

**Keywords:** diabetes mellitus, Jerusalem artichoke, molecular docking, network pharmacology

## Abstract

The effective components and mechanism of Jerusalem artichokes (JAs) in lowering blood glucose were studied through network pharmacology and molecular docking. The active compounds of Jerusalem artichoke were obtained by referring to the literature, and the active compounds were screened. The targets were predicted by the SwissTargetPrediction database, and the disease targets were screened using GeneCard, Disgenet, and OMIM databases. The protein–protein interaction (PPI) network diagram was constructed using the STRING database, and the intersection target was analyzed by gene ontology (GO) biological function and Kyoto Encyclopedia of Genes and Genomes (KEGG) pathway analyses using the David database. Finally, molecular docking was verified using AutoDockTools1.5.7 software. After screening, 412 gene targets, 476 disease targets, and 64 intersection targets were identified. The results of GO biological function analysis and KEGG pathway analysis showed that the technology was involved in multiple biological processes and regulatory pathways for hypoglycemia, such as the HIF-1, PI3K-Akt, and AMPK signaling pathways. Molecular docking results showed that Jasmonate, Liquiritigenin and Inulin of JAs had strong binding effects with PPARG and STAT3. JAs exert hypoglycemic effects through multi-component, multi-target and multi-pathway. In summary, this study investigated the hypoglycemic mechanism of JAs using network pharmacology and molecular interconnection technology, and concluded that JAs exert hypoglycemic effects through multiple components, targets, and pathways, which provides a theoretical basis for the study of JAs.

## 1. Introduction

Diabetes, the most prevalent metabolic disorder, has a profound impact on the global health system. It has become a severe, chronic, noncommunicable disease after cardio-cerebrovascular diseases. Hyperglycemia is the main hallmark of diabetes.^[1]^ According to the latest data released by the International Diabetes Federation, approximately 463 million adults between the ages of 20 and 79 in the world suffered from diabetes in 2019, accounting for 1/11 of the total population, of which 110 million people in China had diabetes, ranking first in the world.^[2]^ It is 1 of the world’s fastest-growing diseases, with 783 million adults expected to be affected by the year 2045. Devastating macrovascular consequences (cerebrovascular disease, cardiovascular disease, and peripheral vascular disease) and microvascular complications (such as retinopathy, nephropathy, and neuropathy) increase mortality, blindness, kidney failure, and the overall quality of life in individuals with diabetes.^[3]^ There is no specific treatment for diabetes, and prevention and control remain the primary treatment options. Western medicines such as metformin tablets and acarbose tablets are clinically used to treat diabetes. Although they have significant efficacy, they have obvious side effects and drug dependence.^[4]^ Scientific studies have shown that the occurrence and development of diabetes is closely related to diet, and the use of medicinal diet therapy to improve diabetes and adjuvant treatment of glycosuria has been increasingly recognized.^[5]^ Therefore, identifying Chinese medicines that can prevent and treat diabetes has become a hot research topic.

Jerusalem artichoke (JA), also known as *Helianthus tuberosus L.*, is a perennial herb belonging to the sunflower family Compositaeand is cultivated in North America, Northern Europe, East Asia, Australia, and New Zealand.^[6]^ JA contains essential amino acids, proteins, minerals, and bioactive and functional components such as oligofructose, inulin, fructose, and flavonoids.^[7]^ JA is a plant of great importance to human and animal nutrition and health. Plant tubers have both functional (medicinal) and nutritional properties and are particularly beneficial for obesity and type 2 diabetes.^[8]^ JA is a perennial tuberous plant with a wide environmental adaptability. JAs have strong resistance to abiotic stresses, low nutrient demand, strong ecological-restoration effects, and high commercial value.^[9]^ At present, the main method to study the JA extract is to prove its effect and mechanism through mouse and cell experiments. However, because of the large number of active substances in JA extract, various mechanisms of hypoglycemic action, and complicated targets, its mechanism remains unclear.^[6,10]^

**Figure 1.**
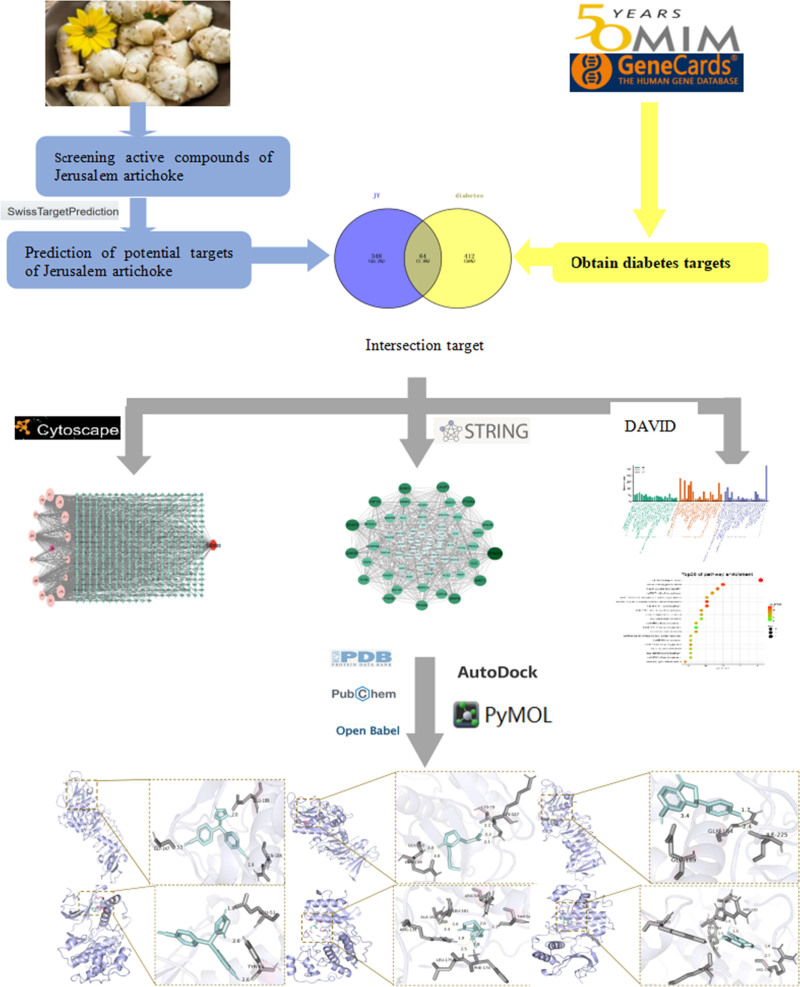
A flowchart of this study.

Network pharmacology combines system network analysis with pharmacology, bioinformatics, and other disciplines to demonstrate the multi-component and multi-target drug treatment processes from the perspective of genes.^[11]^ Traditional Chinese medicine (TCM) has multi-target versatility. Through the study of network pharmacology, the mechanism of action of TCM in the treatment of diseases was clarified, providing new ideas for the development of TCM.^[12]^ In this study, the molecular mechanism of the active ingredients in JAs was studied through network pharmacology and molecular docking technology, which provides a theoretical basis for further study of the hypoglycemic effect of JAs. A flowchart of the study is presented in Figure 1.

## 2. Materials and methods

### 2.1. Screening of effective components of Jerusalem artichoke and collection of component targets

Find and read the literature on JAs to obtain the effective chemical composition of JAs. The CAS number of the chemical composition formula was obtained from the chemical source website (https://www.chemsrc.com/). A map of the chemical materials can be found on the PubChem website (https://pubchem.ncbi.nlm.nih.gov/). The chemical structure was uploaded to the filtered SwissTargetPrediction database (http://www.swisstargetprediction.ch/), and oral bioavailability ≥ 30% and drug-likeness ≥ 0.18 were selected as the criteria for screening, and target targets with probability > 0 were selected as the targets of JA’s active ingredient. The predicted targets were normalized using the UniProt database (https://www.uniprot.org/), which were reviewed and calibrated by species and humans, and then duplicated values were removed to obtain the chemical composition targets of JAs.^[13]^

### 2.2. Collection targets of diabetes

Using “diabetes” as the keyword, OMIM database, GeneCards database, DrugBank data Disease gene databases such as the database were searched and screened respectively, and the obtained targets were summarized and duplicated values were removed. Finally, the obtained targets were standardized using the UniProt database.^[14]^

### 2.3. Identification of common targets and construction of drug–disease–target network

To clarify the relationship between chemical composition and disease targets, the obtained targets were compared and the intersection of the 2 was selected to draw a Venn diagram using Venny2.1.0. Cytoscape3.10.0 software (https://cytoscape.org/) was used to construct a visual network diagram of “drug–disease–target.”

### 2.4. Construction of protein–protein interaction network

The intersection targets of the chemical components of diabetes mellitus and Jerusalem arum were uploaded to the STRING database (https://string-db.org/) to obtain the interaction between proteins. Then Cytoscape3.10.0 software was used to map the protein–protein interaction (PPI) network.

### 2.5. GO biological function analysis and KEGG pathway enrichment analysis

Using the David database (http://David.Ncifcrf.Gov/viewJSP) for GO intersection targets and KEGG enrichment analysis, we performed GO function analysis, including biological process (BP), cell components (CC), molecular function (MF) analysis. The results from KEGG pathway analysis indicate that JA is a key pathway for diabetes.

### 2.6. Molecular docking

The core targets and chemical components were selected for molecular docking analysis. The molecular structure of the chemical composition of Jerusalem artista was obtained from the PubChem database (https://pubchem.ncbi.nlm.nih.gov/) and converted from sdf format to PDB format using Open Babel software. The molecular structures of peroxisome proliferator-activated receptor-γ (PPARG) and STAT3 were downloaded from the PDB database and treated with water and ligand removal. The chemical composition structure file and Artiothura target protein file were imported into AutoDockTools1.5.7 software (https://autodock.scripps.edu/) for docking experiments, and the processing results were saved as pdbqt format file. PyMOL 2.6 software was used to draw the display diagram of the docking results.

## 3. Results

### 3.1. Screening of active ingredients and target genes

After reviewing the literature, the chemical components of Jerusalem artichox were uploaded to the Swiss ADME database for screening. Fourteen compounds with good oral absorbability and chemoid-like properties were selected and numbered JY1–JY14. The corresponding compounds were Inulin, Tagitinin E, Jasmonic acid, sulfurein, Coumarin-3-carboxylic acid, sinapic acid, 4-hydroxybenzoic acid acid, salicylic acid, ferulic acid, liquiritigenin, beta-sitosterol, gallic acid, caffeic acid, and pyrenocin A (Table 1). The 14 chemical structure formulas were successively imported into the SwissTargetPrediction database for prediction, and the predicted targets of related compounds were obtained. The chemical composition targets were normalized using the UniProt database and duplicated items were merged and removed. Finally, 412 chemical composition targets of the JAs were identified.

**Table 1:** The active ingredients of Jerusalem artichokes.

No	Active Ingredients	CAS number
JY1	Inulin	9005-80-5
JY2	Tagitinin E	59979-58-7
JY3	Jasmonic acid	3572-66-5
JY4	Sulfurein	120-05-8
JY5	Coumarin-3-carboxylic acid	531-81-7
JY6	Sinapic acid	530-59-6
JY7	4-Hydroxybenzoic acid	99-96-7
JY8	Salicylic acid	69-72-7
JY9	Ferulic acid	1135-24-6
JY10	Liquiritigenin	578-86-9
JY11	Beta-Sitosterol	83-46-5
JY12	Gallic acid	149-91-7
JY13	Caffeic acid	331-39-5
JY14	Pyrenocin A	76868-97-8

### 3.2. Identification of common targets and construction of the “drug–disease–target” regulatory network

**Figure 2.**
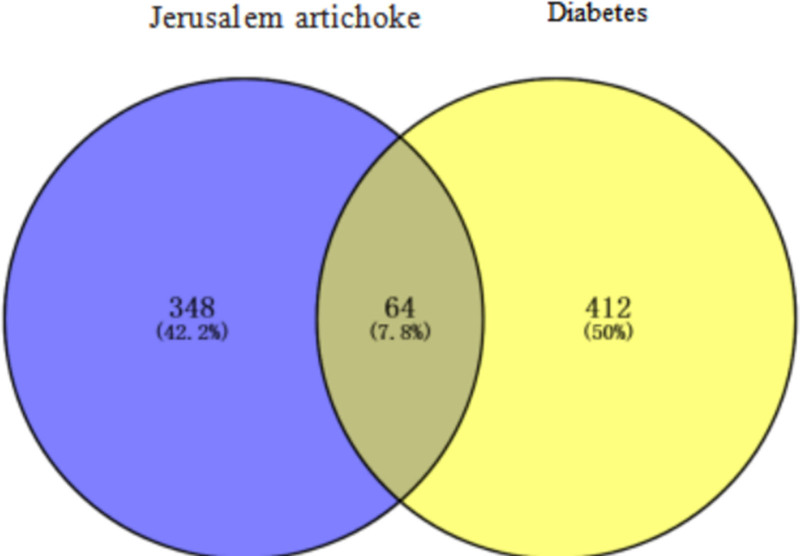
A Venn diagram of the Jerusalem artichoke–diabetes target.

**Figure 3.**
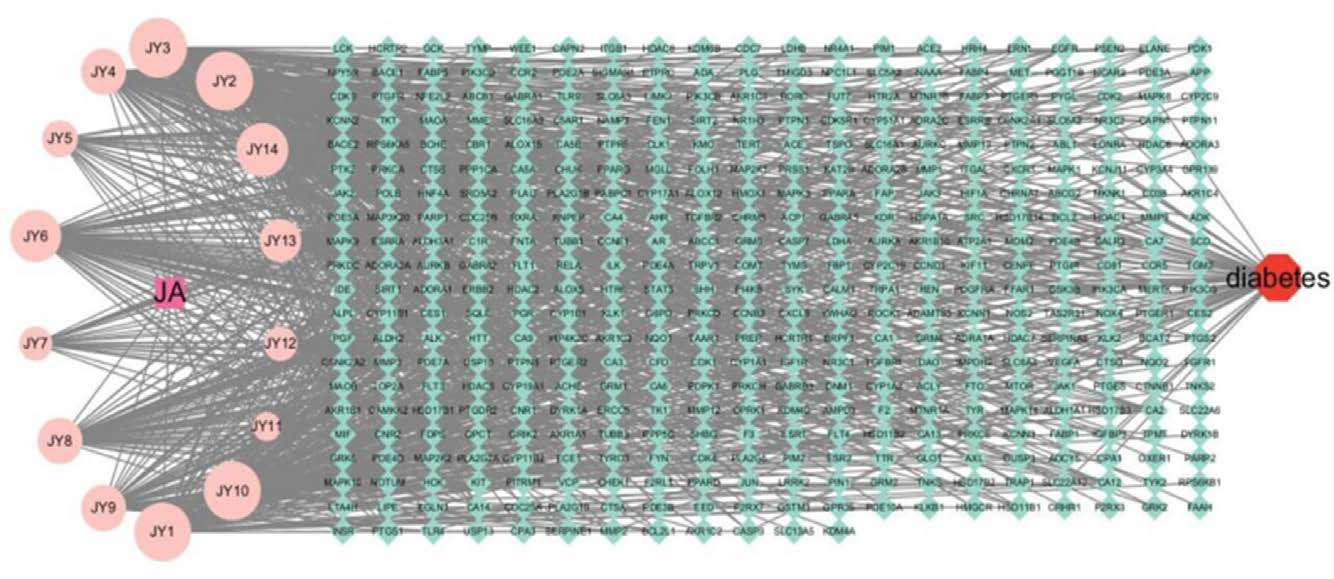
The “drug-disease-target” network of JA in the treatment of JA. Squares represent drugs, circles represent ingredients, and diamonds represent gene targets. JA = Jerusalem artichoke.

By searching and screening in the OMIM, GeneCards, and DrugBank databases, the diabetes disease targets were summarized and duplicate values were removed, and 476 targets closely related to diabetes were obtained. After the intersection of the chemical composition of Jerusalem artixum and diabetes mellitus was obtained using the Venny2.1.0 online mapping tool, 64 targets of the intersection of Jerusalem artixa-disease were obtained, accounting for 7.8% of the total number of targets, and a Wayne diagram of Jerusalem artixa-disease was plotted (Fig. 2). Cytoscape 3.10.0 software (https://cytoscape.org/) was used to construct the relationship network of “drug-disease-target” of Jerusalem artixum (Fig. 3). The network comprises 924 nodes and 3913 edges. The degree of the network represents the number of edges connected to a node. A higher degree value of a component or target suggests its potential as a key component or target.^[15]^ The “drug-disease-target” network diagram clearly and intuitively shows the network diagram of a chemical component of JAs with multiple targets for the treatment of diabetes. The results showed that JAs acted on multiple targets through the action of multiple components, and the complex network diagram proved the potential role of JAs in the treatment of diabetes.

### 3.3. Construction of protein–protein interaction network

**Figure 4.**
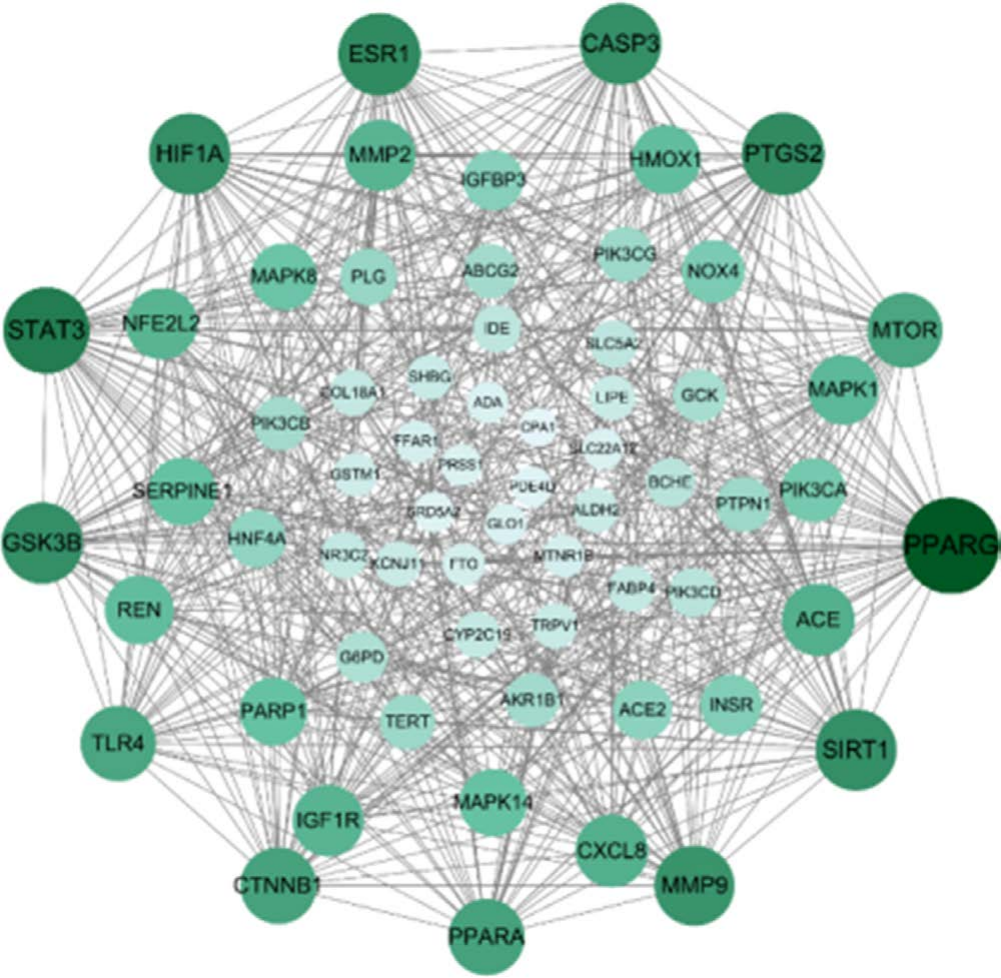
The PPI network of JA in treating diabetes. JA = Jerusalem artichoke, PPI = protein–protein interaction.

64 intersection targets were imported into the STRING database for the PPI network analysis. The PPI network diagram of intersection targets showed 64 nodes with 559 edges, average node degree of 17.5, and average local clustering coefficient of 0.662. The .tsv file was obtained from the STRING database and imported into Cytoscape3.10.0 software to map the PPI network. As shown in Figure 4, the larger the circle and the darker the color in the figure, the larger the degree value, which is proportional to the size of the node. The larger the node, the more important is the target corresponding to the node in the PPI network.^[16]^ A network analyzer was used to evaluate the network topology characteristics, sorted according to the degree value of nodes, and 8 core nodes were subsequently determined with a degree greater than twice the average of the PPI network.^[17]^ As shown in Figure 4, the targets highly associated with diabetes included PPARG, STAT3, ESR1, and so on.

### 3.4. *Gene ontology biological function annotation and KEGG pathway* enrichment analysis

**Figure 5.**
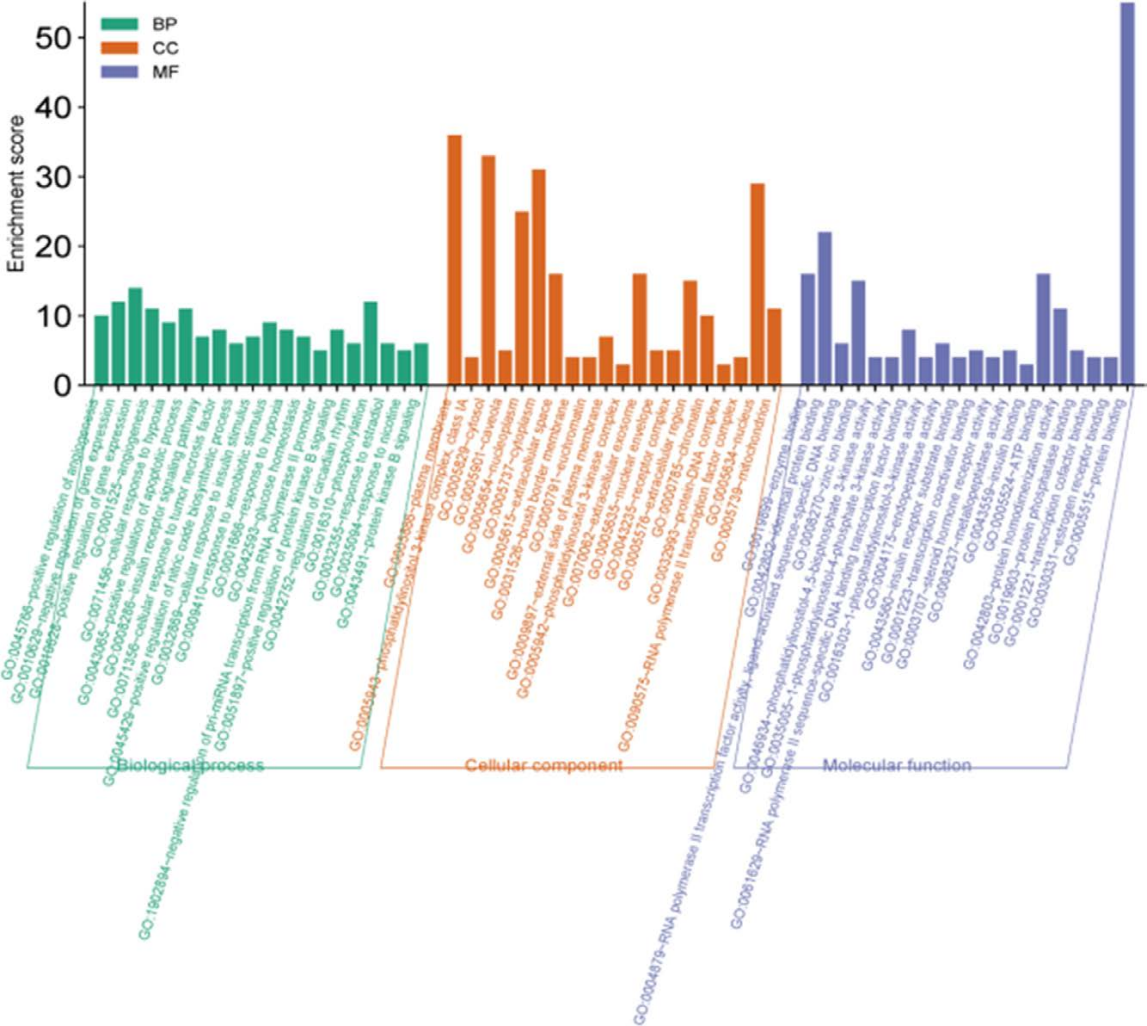
Top 20 significant GO enrichment entries of Jerusalem artichoke in treating diabetes. GO = gene ontology.

The selected 64 intersection targets were introduced into the David database for GO biological function and KEGG pathway enrichment analyses. The results were screened with a *P*-value < .05.^[18]^ The analysis results showed that the 64 intersection targets involved 310 cellular BPs, such as positive regulation of angiogenesis, angiogenesis, cell response to hypoxia, insulin receptor signaling pathway, cell response to tumor necrosis factor, positive regulation of nitric oxide biosynthesis, and cell response to insulin stimulation. Forty CC, such as plasma membrane, phosphatidylinositol 3-kinase complex, Class IA, cytosol, etc 72 MFs, such as enzyme binding, phosphatidylinositol-4, 5-diphosphate 3-kinase activity, 1-phosphatidylinositol-4-phosphate 3-kinase activity, 1-phosphatidylinositol-3-kinase activity, endopeptidase activity, and insulin receptor substrate binding. Based on the *P*-value ranking selection of BP, MF, CC the top 20 were analyzed, and the use of microscopic letter platform (http://www.bioinformatics.com.cn/) will GO biological visual function analysis results, top 20 significant GO enrichment entries of JA in treating Diabetes are shown in Figure 5.

**Figure 6.**
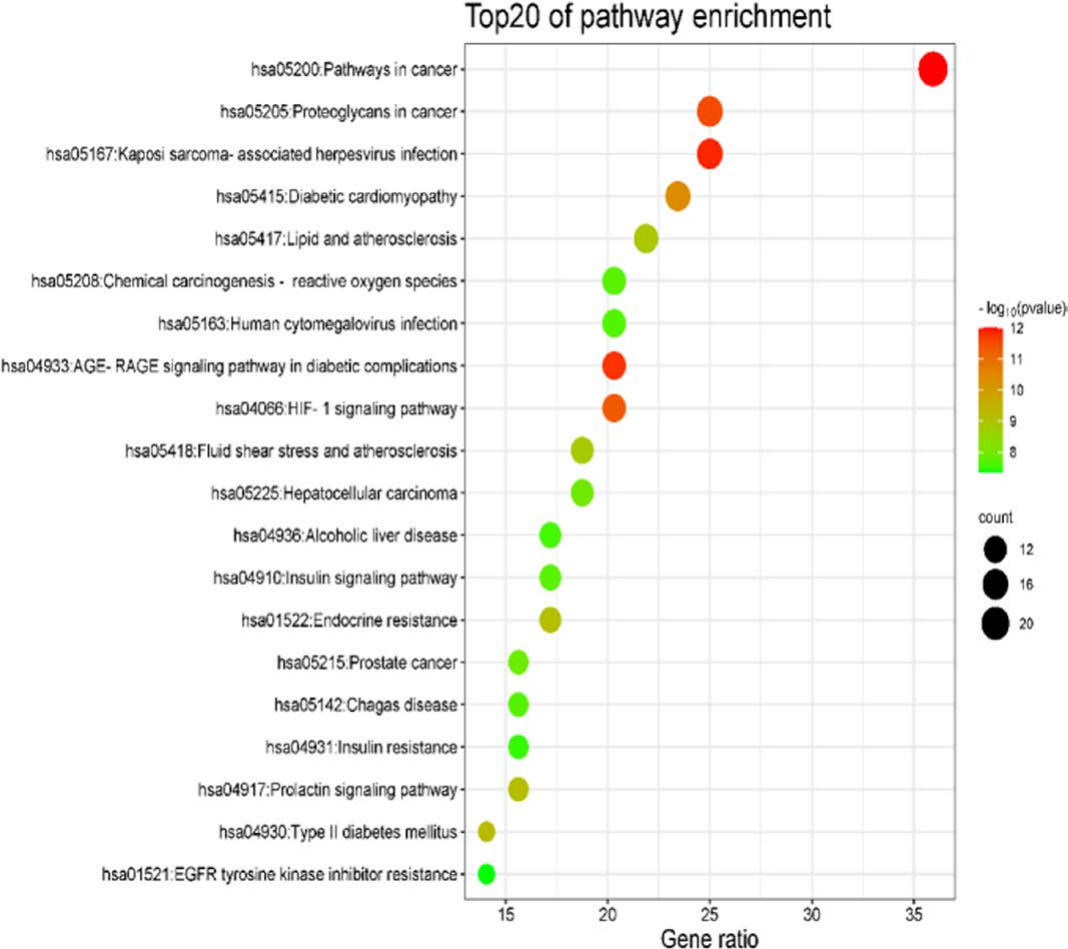
Top 20 significant KEGG enrichment entries of JA in treating diabetes. JA = Jerusalem artichoke, KEGG = Kyoto Encyclopedia of Genes and Genomes.

Analysis of the Kyoto Encyclopedia of Genes and Genomes (KEGG) pathway of the chemical composition of JA and the intersection target of diabetes mellitus, 134 pathway data were obtained. There are several signaling pathways related to hypoglycemia, such as the HIF-1 signaling pathway, insulin signaling pathway, PI3K-Akt signaling pathway (PI3K-Akt signaling pathway), insulin resistance, AMPK signaling pathway, and type II diabetes mellitus (type II diabetes). The top 20 channel data according to the size of the *P*-value used to draw the bubble diagram are shown in Figure 6. The HIF-1 signaling pathway in the figure contains 13 targets; the insulin signaling pathway contains 11 targets; the PI3K-Akt signaling pathway contains 11 targets; the insulin resistance pathway consists of 10 points; AMPK signaling pathway contains 10 targets; the Type 2 diabetes pathway contains 9 targets.^[19]^

Through the analysis of GO biological function and KEGG pathway enrichment results, it was found that the chemical components of JA play a role in the treatment of diabetes through multi-target, multi-component and multi-pathway.

### 3.5. *Molecular docking result*

**Figure 7.**
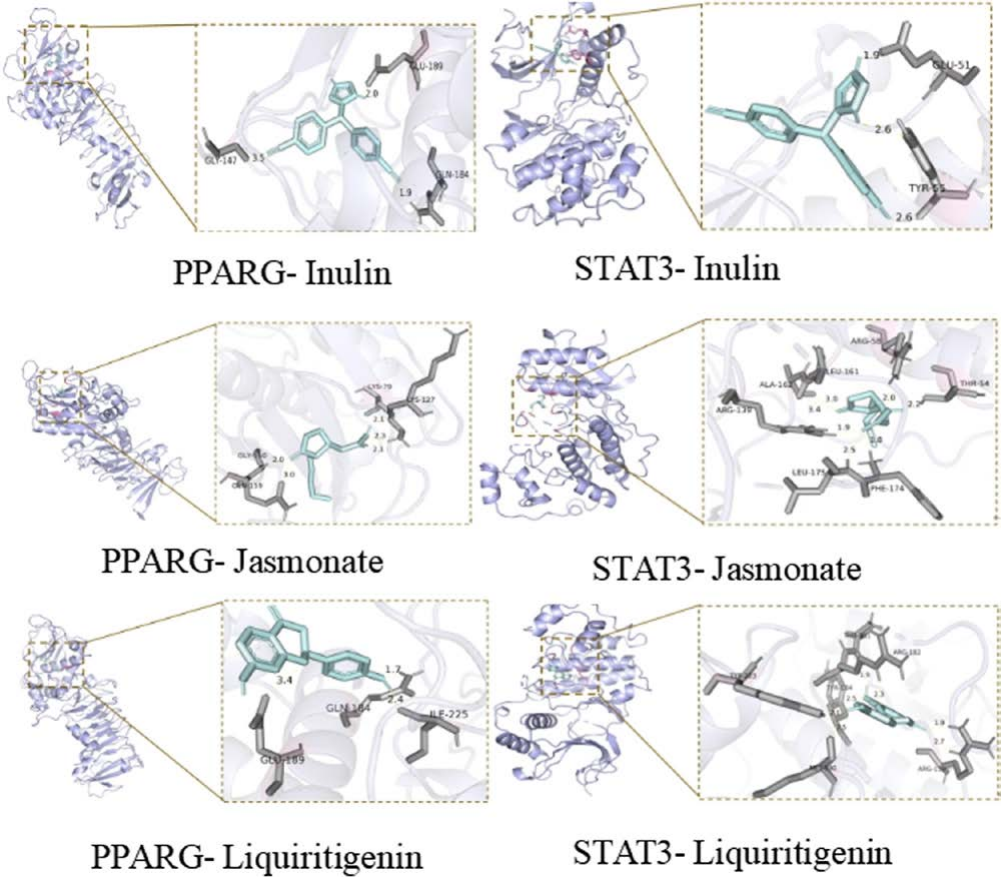
The results of molecular docking of the main components and core targets.

The chemical components JY3 (jasmonate), JY10 (liquiritigenin), and JY1 (inulin), which ranked among the top 3 in the degree of core target PPARG, STAT3, and artichokes, were selected for molecular docking using AutoDockTools 1.5.7. Molecular docking binding energies are presented in Table 2. The binding energies between 2 molecules can predict their binding activity. When the binding energy is negative, the 2 molecules can spontaneously react. The smaller the binding energy, the more stable the binding of the 2 molecules.^[20]^ When the binding energy is less than −5.0 kcal/mol, the 2 molecules have good binding activity; when the binding energy is less than −7.0 kcal/mol, it indicates that the binding activity between the 2 molecules is very good.^[21,22]^ As shown in Table 2, the junction and energy of all molecular docking are negative, and the binding energy of PPARG-JY3 (Jasmonate) is −5.07 kcal/mol, which is less than −5.0 kcal/mol, indicating that the binding activity between the 2 molecules is good. The binding energies of PPARG-Inulin, PPARG-Liquiritigenin, STAT3-Jasmonate, STAT3-Liquiritigenin and STAT3-Inulin were all lesser than −7.0 kcal/mol, indicating strong binding energies between core targets and effective components of artichokes. The binding sites were visually analyzed and mapped using PyMOL software (Fig. 7). The results showed that the key active components of JAs could form stable hydrogen bonds and binding conformations with the core target protein. The results of molecular docking indicated that the active ingredients of Jerusalem artista might be combined with PPARG and STAT3 to treat diabetes, and the results of molecular docking further proved the accuracy of the network pharmacological screening.

**Table 2:** Docking results between key compounds and key targets (unit: kcal/moL).

Compounds	Targets	
	PPARG	STAT3
Inulin	−7.45	−7.07
Jasmonic acid	−5.07	−7.63
Liquiritigenin	−7.33	−8.85

Abbreviations: CAS = chemical abstracts service, PPARG = peroxisome proliferator-activated receptor gamma, STAT3 = signal transducer and activator of transcription 3.

## 4. Discussion

In this study, the mechanism of JAs in the treatment of diabetes was analyzed using network pharmacology and molecular docking. Fourteen effective components of Jerusalem artichox were obtained after screening, comprising 412 targets. Through the analysis of the visual relationship network diagram of chemical composition, targets, and diabetes mellitus, it was found that Jerusalem artichox has the characteristics of multiple components and targets for lowering blood glucose. The core targets were screened using a PPI network map, among which PPARG, STAT3, and other core targets played an important role in the hypoglycemic mechanism of Jerusalem arum.

The results of GO biological function analysis showed that the chemical components of JA in the treatment of diabetes were mainly involved in the positive regulation of angiogenesis, insulin receptor signaling pathway, cell response to insulin stimulation, and other BPs. According to the results of KEGG pathway analysis, the hypoglycemic effect of the chemical components of Jerusalem arum mainly involves the HIF-1 and PI3K-Akt signaling pathways. Among these, the HIF-1 signaling pathway regulates cell perception and adaptation to internal environmental changes. When organisms are in a state of high glucose or hypoxia, the HIF-1 signaling pathway can promote an increase in the anaerobic fermentation process and induce the expression of downstream inflammatory response-related genes.^[23]^ The PI3K-Akt signaling pathway contains 11 targets. Insulin plays an important role in the regulation of blood sugar level in the human body, and the PI3K-Akt signaling pathway is the main pathway of insulin signal transduction.^[24]^ Therefore, it is speculated that the chemical components of JA treat diabetes mainly by regulating the HIF-1 and PI3K-Akt signaling pathways.

Molecular docking results showed that PPARG and STAT3 had strong binding with jasmonate, liquiritigenin, and inulin from JA, and PPARG is a transcription factor belonging to the nuclear hormone receptor superfamily. PPARG expression was detected mainly in the adipose tissue. Previous studies have indicated a significant role of PPARG and *PPARG* gene polymorphisms in the pathogenesis of type 2 diabetes^[25]^; STAT3 can regulate the expression of genes related to cell survival, proliferation, activation, and differentiation, and plays an important role in islet development and insulin secretion.^[26]^ The chemical components of JAs may combine with PPARG, STAT3, and other targets, act on insulin synthesis and other processes, and play a role in lowering blood sugar.

In summary, this study investigated the hypoglycemic mechanism of JAs using network pharmacology and molecular interconnection technology, and concluded that JAs exert hypoglycemic effects through multiple components, targets, and pathways, which provides a theoretical basis for the study of JAs.

## Acknowledgments

The authors thank MS Yijie Han for her invaluable assistance with the data acquisition and bioinformatic analysis. We would like to thank the researchers and study participants for their contributions.

## Author contributions

**Conceptualization:** Xueting Zhu.

**Data curation:** Xueting Zhu.

**Formal analysis:** Xueting Zhu.

**Methodology:** Zhihua Guo.

**Project administration:** Zhihua Guo.

**Software:** Xueting Zhu.

**Visualization:** Xueting Zhu.

**Writing – original draft:** Zhihua Guo.

**Writing – review & editing:** Xueting Zhu.
